# Latent and Asymptomatic* Toxocara* Infection among Young Population in Northwest Iran: The Necessity of Informing People as a Potential Health Risk

**DOI:** 10.1155/2016/3562056

**Published:** 2016-02-28

**Authors:** Tina Momeni, Mahmoud Mahami-Oskouei, Esmaeil Fallah, Abdolrasoul Safaiyan, Leyla Mahami-Oskouei

**Affiliations:** ^1^Immunology Research Center, Tabriz University of Medical Sciences, Tabriz, Iran; ^2^Department of Parasitology and Mycology, Faculty of Medicine, Tabriz University of Medical Sciences, Tabriz, Iran; ^3^Department of Biostatistics and Epidemiology, Faculty of Health, Tabriz University of Medical Sciences, Tabriz, Iran

## Abstract

*Objectives*. This study was designed to determine the frequency of anti-*Toxocara* antibodies in youngsters aging from 2 to 20 years in northwest Iran.* Materials and Methods*. 397 samples were taken randomly almost equally from four locations in Urmia, west Azerbaijan, during August 2014 to September 2015. Anti-*Toxocara* IgG antibody assays were done on sera by using ELISA kit (IBL, Germany). In order to prevent cross-reaction, the samples of the patients who are infected with other parasites in stool exam, especially Ascarididae family, were also excluded. SPSS 16.0 software was used for statistical analysis.* Results*. 12 (3%) of the serum samples were positive for anti-*Toxocara* IgG. According to the Chi-square analysis, risk factors such as mother's educational level, keeping dogs or cats as pets, and history of coughing were related to* Toxocara* infection (*P* < 0.05). There was no relationship between toxocariasis and gender, history of onychophagy, pica, fever, abdominal pain, and anorexia; however, we found a significant relationship between* Toxocara* infection and chronic coughing (*P* = 0.045).* Conclusion*. Toxocariasis in northwest Iran can be considered as a public health problem. This study may also help to increase the awareness about this infection.

## 1. Introduction

Human toxocariasis is one of the canine parasitic infections caused by the larvae stage of the genus* Toxocara*. This disease was first described in the 1950s and was long considered an abnormal illness among children. This parasite, which has global distribution, may be found in both mankind and animals. The prevalence rate varies from one location to another. Newfound information, concerning this parasite, led to knowledge of the various syndromes caused by this infection [1, 2]. Clinical signs and complications which result from infection with this parasite are mostly dependent on the number and migration locations of* Toxocara* larvae, mainly categorized to three groups: migrating visceral larvae, ocular larvae, and covert form. In human, asthma, loss of vision, hypereosinophilia, encephalitis, and problems involving the liver, lungs, and the CNS are the most important complications caused by this parasite [3]. Previous investigation indicated a relationship between epileptic seizures and infection with various types of* Toxocara* parasite [4]. In addition, a study performed about ocular toxocariasis in order to introduce better diagnosis and treatment showed that this parasite is still affecting many children and causing many complications, such as permanent blindness in the patients [5]. Numerous factors such as life style, environmental contamination, keeping pets, and economic and social parameters are also important in the prevalence of* Toxocara* infection in human [6, 7]. A study performed in Tabriz, Iran, showed that most of pet owners had a high contact risk with this parasite [8]. Different studies have been conducted to determine the relevance between the serum prevalence of toxocariasis and hypereosinophilia; according to the results, such a relation does indeed exist [9–11]. For example, anti-*Toxocara* antibodies and their relation with the age, sex, and peripheral eosinophils in lymphatic filariasis patients have been studied in northeastern Brazil [12]. Many investigations have been performed in various regions for the detection of* Toxocara* eggs from soil sources and also the determining of the presence of anti-*Toxocara* antibodies in their residents. Recently, similar studies have also been performed in Iran to determine the infection rate of* Toxocara* in canine [13–15]. Seroepidemiological analysis of toxocariasis infection among children was done in Zanjan; the results indicate that the infection rates were lower in comparison to other cities. Increasing the hygiene levels is considered to be a paramount factor in the controlling of this infection [16]. Based on the only study conducted in Urmia, west Azerbaijan that solely aimed to determine the levels of* Toxocara* egg in the soil of parks, a contamination rate of 7.8% was reported [17]. In spite of the fact that some seroepidemiological studies have been performed in different regions of Iran and also considering the high contamination rate of the soil of the Urmia parks [17], the serum prevalence of toxocariasis remains as an unresolved problem in this region. Accordingly, the present study was designed for the first time to determine the frequency of anti-*Toxocara* antibodies in youngsters aging from 2 to 20 years in Urmia, northwest Iran. The results and findings of this study will be made available for other researchers, as well as health officials in the hope of helping them to better evaluate the prevalence and distribution of this parasite in the northwestern regions of the country, especially west Azerbaijan.

## 2. Materials and Methods

In this cross-sectional study, 397 samples were taken randomly from 2 to 20 years almost equally from four locations in Urmia during August 2014 to September 2015. To increase the reliability of the tests, hyperlipidaemic and haemolysed samples were removed from the study. In order to prevent cross-reactions between* Toxocara* larvae and other organisms' antigens, stool exam by using wet mount and formalin-ethyl acetate technique was also performed and patients who are infected with the other parasites in stool exam, especially Ascarididae family, were excluded. Age and gender ratios were kept constant between the regions. This study has been approved by the ethics committee of Tabriz University of Medical Sciences, Iran. After receiving a written informed consent, a questionnaire including age, sex, education level, keeping dogs and/or cats, geophagy, onychophagy, fever, chronic coughing, abdominal pain, and anorexia was filled for each person along with sampling. In the present investigation, we also measured some hematological parameters such as HCT, Hb, and ESR values and PLT, RBC, and WBC count. After collecting about 2–2.5 mL of the whole blood samples in tubes and preparing blood smear in order to confirm eosinophilia, serums were separated and stored at −20°C. Anti-*Toxocara* IgG antibody assays were done on sera by* T. canis* excretory-secretory (TES) antigens using ELISA kit (IBL, manufactured in Hamburg, Germany) according to the manufacturer's instructions. SPSS 16.0 software was used for statistical analysis. Quantitative and qualitative parameters were evaluated by independent sample *t*-test and Chi-square, respectively. Finally *P* value < 0.05 was considered as significant level.

## 3. Results

From a total of 397 serum samples, 12 (3%) were positive for anti-*Toxocara* IgG by using ELISA technique. The other 385 (97%) samples were negative. The infection rate in female and male was 1.4% and 4.8%, respectively. There was no significant relationship between gender and* Toxocara* infection rates (*P* = 0.054). 10.3% of seropositive individuals had a history of keeping pets especially cat or dog. Hence, in this study, a significant relationship was observed between pet held and infection with the parasite (*P* = 0.003). In terms of clinical signs, 5.7%, 5.9%, 4.8%, 6.1%, 4.7%, and 3.5% of the patients had onychophagy history, pica, fever, chronic coughing, abdominal pain, and anorexia, respectively. There was no statistical significant relationship between toxocariasis and history of onychophagy, pica, fever, abdominal pain, and anorexia; however we found a significant relationship between* Toxocara* infection and chronic coughing (*P* = 0.045). Although, education level of seropositive cases and father of the patients had no impact on the disease in this investigation, but the patient's mother's education level has been effective to the rate of infection (*P* = 0.042). Hematologic parameters in* Toxocara* positive and negative cases are described and compared (Table 1). Quantitative analysis showed that an increase in the numbers of peripheral eosinophils, which were counted using the prepared blood smears, was seen in the sample of the patients whose serum was positive for anti-*Toxocara* antibodies. In addition, the ES rate of positive samples was about 2.3 times of IgG negative samples. This increase is indicative of inflammation in patients with* Toxocara*. In this study, stool exam was also performed along with serological evaluation. After sedimentation by using formalin-ethyl acetate technique, all samples were examined microscopically. The results showed that overlay 26 (6.55%) of samples were infected with different parasites that among them three seropositive samples were also infected with the other parasites (Table 2). In the present study, we did not see any egg of* Ascarididae* family parasites.

## 4. Discussion

Seroepidemiological studies in different countries have shown the global distribution of toxocariasis. In developed countries, visceral larvae migrans is considered to be the second most common worm infection in human. Although in developing countries other worm infections are more common, but reports indicate high infection rates by* Toxocara* larvae [14, 15]. In the present study, we detected anti-*Toxocara* antibody in 3% of samples. According to the results, seropositive patients had significantly higher eosinophilia in comparison with seronegative cases, indicating a relationship between toxocariasis and hypereosinophilia. In the previous studies, hypereosinophilia was also reported to relate to* Toxocara* infection [18]. For example, in a study performed in Chungcheongnam, South Korea, on hypereosinophil patients, it was observed that toxocariasis is related to the condition, which may have been the result of consuming raw cow liver and play a significant role in the transmission of the parasite [10]. Another study was performed with the aim of determining the serum prevalence of anti-*Toxocara* antibodies in children with chest wheezes in Tehran. The results indicate that children who were attending school had the highest prevalence of infection in comparison with the other groups who have had hypereosinophilia and allergic reactions [11]. In a study conducted by Kim et al. on the adult population of Seoul, amongst the 97 patients who were studied, 63 (65%) had anti-*Toxocara* antibody [19]. Also, in a study performed in Shiraz, southern Iran, on children aged 6 to 13 years, prevalence rate was reported to be about 25.6%. Amongst those infected, 20.2% resided in rural areas, while 30.1% were city residents. The results of this study vary from that of those conducted in other regions which is said to be related to the high infection prevalence of the cats and dogs with parasites such as* T. cati* and* T. canis* [20]. In other previous studies that were performed in Iran and Turkey, no relationship was observed between the infection rate and children residency [21, 22]. Different prevalence in the studies may be the result of many factors such as more contact with infected cats and dogs in rural regions. In addition, the contact prohibition with canines and felines varies in different religions. For example, in Islam, it is strictly forbidden to come in contact with dogs, or any other material for that matter, which has been in any way exposed to the animal itself, or its urine and faeces. On the other hand, kits and the cut-off used in data analysis may also be different from one study to another. In the present study, according to the Chi-square analysis, no significant relationship was observed between toxocariasis and onychophagy, education level, gender, abdominal pain, fever, and anorexia (*P* < 0.05). Fallah et al. also conducted a study in western Iran, on children aging from 1 to 9 years; no relevance was seen between age, sex, and area of residency. According to the study carried out in Sparta, southwestern Turkey, environmental pollution and life style strongly affect the epidemiology of toxocariasis [7]. The prevalence of toxocariasis was studied in three of the city outskirts in Campinas, Brazil; the results showed the significant effect of socioeconomic parameters on human infection rates [6]. These researches and their findings are consistent with the results of the present study. In a seroepidemiological study which was conducted in Iran, no significant relationship was observed between infection rate and age/gender [23, 24]. In addition, the study performed in Tabriz determined that the gender is irrelevant to the presence of anti-*Toxocara* antibodies (*P* = 0.275) [8]. The results of both studies are consistent with that of ours. Contrary to our findings, in some studies, the relevance between gender and infection rate was reported to be significant and meaningful. For example, in a study performed in Trinidad, infection rates were considerably higher in boys when compared to girls [13]. In northern Jordan, significant differences were seen in infection rates between girls and boys; this difference is mostly related to the games and habits of young boys [25]. Although the infection rate in this study is higher in boys than girls, this difference is of no analytical value and significance. According to the Chi-square analysis, the frequency of infection was related to mother's education level and area of residence. This may be due to culture and behavioural attributes concerning the contact between their child and infected soils. Based on the findings of this study, chronic coughs are also related to the infection rate.

Although in some studies the relationship between contact with dogs or cats and larva migrans syndrome has been reported, other researchers have not observed such relevancy. In a study that was performed in Tabriz, a significant relationship was reported between contact with cats or dogs and* Toxocara* infection rate [8]. But, in contrast, in a study performed by Alavi et al., significant relation was not seen between the prevalence of toxocariasis and contact with dogs which may be due to religious and cultural beliefs [9, 23]. According to a study that is aiming to determine the relevance between* Toxocara canis* infection and raw cow liver consumption, keeping dogs and consuming raw cow liver significantly increase infection rate [26]. According to some investigations, although the natural host of* Toxocara* parasites is mainly canines and felines, the risk of coming in direct contact with these animals is not exponentially high. This is because the eggs are usually discharged through faeces and require at least two week to reach noticeable infectivity. However, dogs that are kept in the same environmental conditions are considered a constant risk for migrating larvae [27].

Different serological methods are available for detecting anti-*Toxocara* antibody in human serum. Among these methods, IFA test is capable, useful and sensitive but this method requires a highly laboratory equipment and trained personnel and also there is no commercially standardization for this purpose. In comparison, ELISA method is much simple, with easier performance and does not require any complicated equipment. It has commercially available kit with appropriate sensitivity and specificity and it is recommended as effective and useful method in seroepidemiological studies of human toxocariasis [28, 29]. In the present study, we used ELISA kit to recognize anti-*Toxocara* IgG antibody in serum samples and also stool exam was performed to prevent cross-reactions.

## 5. Conclusion

Toxocariasis in northwest Iran can be considered as a public health problem. The clinical signs especially chronic coughs, hypereosinophilia, hepatomegaly, or nonspecific pulmonary diseases may be confused with toxocariasis diagnosis. It is necessary for physicians to consider the clinical symptoms of this infection and its similarity with other diseases. This study may also help us to increase the awareness about this infection, as well as having an impact on the care which veterinarians should take in treating intestinal worms. Most of all, this study emphasizes the need of controlling stray dogs in the environment.

## Figures and Tables

**Table 1:** Parameters in *Toxocara* positive and negative cases.

Parameter	Seronegative ($\overset{-}{x}\pm SE$)	Seropositive ($\overset{-}{x}\pm SE$)	*P* value
WBC	8.3 ± 0.19	8.44 ± 1.26	0.904
RBC	4.68 ± 0.03	4.63 ± 0.17	0.826
ESR	18.7 ± 0.88	42.0 ± 9.85	0.000
Hemoglobin	12.49 ± 0.07	12.54 ± 0.44	0.914
Hematocrit	37.95 ± 0.19	38.05 ± 1.17	0.926
Platelets	282.18 ± 5.17	287.83 ± 31.02	0.850
Neutrophils	54.34 ± 0.87	59.83 ± 5.16	0.275
Lymphocytes	37.05 ± 0.81	29.42 ± 5.64	0.106
Monocytes	4.95 ± 0.19	6.25 ± 1.44	0.795
Eosinophils	2.50 ± 0.11	4.50 ± 1.07	0.002
Basophiles	0.09 ± 0.02	0.0 ± 0.0	0.086

**Table 2:** Parasites found in samples after stool examination by using wet mount and formalin-ethyl acetate technique.

Parasite	Seronegative	Seropositive
Giardia lamblia	11	2
Blastocystis hominis	5	1
Entamoeba histolytica	4	0
Oxyur eggs	2	0
Dicrocoelium eggs	1	0
Total	23	3
